# Diabetes Upregulates Oxidative Stress and Downregulates Cardiac Protection to Exacerbate Myocardial Ischemia/Reperfusion Injury in Rats

**DOI:** 10.3390/antiox9080679

**Published:** 2020-07-29

**Authors:** Chen-Yen Chien, Ting-Jui Wen, Yu-Hsiuan Cheng, Yi-Ting Tsai, Chih-Yao Chiang, Chiang-Ting Chien

**Affiliations:** 1 Department of Surgery, Mackay Memorial Hospital, Taipei 10449, Taiwan; ccywyt@msl.mmh.org.tw; 2 Department of Medicine, Mackay Medical College, New Taipei City 25245, Taiwan; 3 Mackay Junior College of Medicine, Nursing and Management, New Taipei City 11260, Taiwan; 4 Department of Life Science, School of Life Science, National Taiwan Normal University, Taipei 11677, Taiwan; J24866489@yahoo.com.tw (T.-J.W.); chanel384305@gmail.com (Y.-H.C.); 5 Division of Cardiovascular Surgery, National Defense Medical Center, Taipei 11490, Taiwan; cvsallen@ndmctsgh.edu.tw; 6 Division of Cardiovascular Surgery, Heart Center, Cheng Hsin General Hospital, Taipei 11220, Taiwan

**Keywords:** diabetes, myocardial ischemia/reperfusion, oxidative stress, apoptosis

## Abstract

Diabetes exacerbates myocardial ischemia/reperfusion (IR) injury by incompletely understood mechanisms. We explored whether diabetes diminished BAG3/Bcl-2/Nrf-2/HO-1-mediated cardioprotection and overproduced oxidative stress contributing to exaggerated IR injury. Streptozotocin-induced diabetes enhanced hyperglycemia, cardiac NADPH oxidase p22/p67 expression, malondialdehyde amount and leukocyte infiltration, altered the mesenteric expression of 4-HNE, CaSR, p-eNOS and BAG3 and impaired microvascular reactivity to the vasoconstrictor/vasodilator by a wire myography. In response to myocardial IR, diabetes further depressed BAG3/Bcl-2/Nrf-2/HO-1 expression, increased cleaved-caspase 3/poly(ADP-ribose) polymerase (PARP)/TUNEL-mediated apoptosis and exacerbated IR-induced left ventricular dysfunction characterized by further depressed microcirculation, heart rate, left ventricular systolic pressure and peak rate of pressure increase/decrease (±dp/dt) and elevated left ventricular end-diastolic pressure (LVEDP) and Evans blue-2,3,5-triphenyltetrazolium chloride-stained infarct size in diabetic hearts. Our results implicated diabetes exacerbated IR-induced myocardial dysfunction through downregulated BAG3/Bcl-2/Nrf-2/HO-1 expression, increased p22/p67/caspase 3/PARP/apoptosis-mediated oxidative injury and impaired microvascular reactivity.

## 1. Introduction

The risk of cardiovascular disease, including myocardial infarction and the overall mortality, is two-fold to four-fold higher in diabetic patients than in non-diabetic ones [1,2]. In addition, diabetics have a lower prognosis than non-diabetics after cardiovascular events [3]. In clinical observation, diabetic patients tend to suffer worse outcomes following cardiac surgery or myocardial ischemia/reperfusion (IR) injury [4], which may in part be related to the altered molecular mechanism in cardiomyocytes or vasomotor and endothelial dysfunction in arteries and arterioles. It is suggested that diabetes enhances oxidative stress in vascular beds and/or cardiomyocytes may contribute to the development and progression of ischemic heart disease [5]. On the other hand, diabetes abrogates cardiovascular protection through the impaired defense mechanism [6] and increases the risk of cardiovascular diseases [7], acute myocardial infarction [8] and mortality [9].

Diabetes-induced oxidative stress may contribute to endothelial dysfunction through a decrease in vasodilating factor nitric oxide (NO) availability and increase in vasoconstricting factor endothelin-1 amount [10], leading to susceptibility to coronary arterial dysfunction. Diabetes can also cause endothelial cells and mitochondria in the cardiomyocytes to produce excessive reactive oxygen species (ROS) production [11] and inflammatory responses resulting in diabetic cardiomyopathy [12]. The greatly increased plasma LDH release and myocardial isoprostane and the depressed plasma superoxide dismutase in diabetes may further increase the cardiomyocyte susceptibility to myocardial IR injury [13]. In response to myocardial injury, diabetes caused a larger infarct size accompanied by decreased cardiac protection of phosphorylated Phosphoinositide 3-kinase (PI3K)/Akt and Janus kinase 2 (JAK2)/STAT3 and cardiac NO levels and these parameters were reversed by antioxidants treatment [14]. Diabetic IR injury depressed cardiomyocyte sirtuin1 expression and activity and Akt activation, leading to mitochondrial impairment and ROS production [15]. One previous report stated that diabetes increased myocardial IR injury-induced oxidative stress and impaired cardiac protection associated with the enhancement of cardiomyocyte caspase 3 activity and decrease in B-cell lymphoma 2 (Bcl-2) expression [13]. Furthermore, diabetes evoked inflammation and oxidative stress possibly via the modulation of the NF-E2 p45-related factor 2 (Nrf-2)/heme oxygenase-1 (HO-1) and NF-κB signaling pathways in diabetic cardiomyopathy [16]

Bcl-2-associated athanogene 3 (BAG3) is highly expressed in the heart and vasculature, interacting with Bcl-2 to inhibit apoptosis, and maintaining the structural integrity of the sarcomere by linking the filament with the Z-disc [17]. BAG3 targets misfolded proteins for lysosomal degradation via the autophagy pathway [18] and mutations in BAG3 have resulted in proteotoxic stress and severe cardiomyopathy [19]. BAG3-deficient animals developed left ventricular dysfunction and early lethality and a fulminant myopathy characterized by noninflammatory myofibrillar degeneration with apoptotic features, whereas depressed *bag3* expression in C2C12 myoblasts increased apoptosis [20]. In cardiac cells, BAG3 plays a critical role in regulating mitochondrial dynamics and mitochondrial quality control [4,21]. Suppressed BAG3 decreased mitophagy and disturbed damaged mitochondrial clearance triggering cardiomyocyte apoptosis induction [21]. BAG3 mutation led to mitochondrial fragmentation and alterations of mitochondrial fission and fusion proteins in hearts leading to progressive heart failure [4]. Overexpressed BAG3 efficiently improved myocardial infarction induced left ventricular dysfunction [17], decreased infarct size and improved markers of autophagy and apoptosis [12]. In the study from Carrizzo et al. [22], they demonstrated that BAG3 exerts its vasorelaxant effect on resistance vessels, especially involved in blood pressure regulation, through activation of the PI3K/Akt signaling pathway, leading to endothelial nitric oxide release. However, it is still uncertain whether the protective role of BAG3 was affected in response to diabetes or myocardial IR injury. Ca^2+^-sensing receptor (CaSR) existed in endothelial and smooth muscle cells and the dysregulated CaSR function and expression may predispose to the macrovascular late complications associated with diabetes [23]. However, the effect of diabetes on CaSR function and expression in regulating vascular reactivity is unknown. Recently, reductive stress, defined as an excess of defense antioxidants aimed at fighting ROS, has emerged as harmful to biological systems for reducing the beneficial roles of ROS on baseline functions, the accomplishment of the immune response during the course of infection and tissue injury and vasodilation [24]. To explore the possible role of reductive stress in diabetes and myocardial IR injury, we thus determined several defense antioxidants including BAG3, Bcl-2, Nrf-2 and HO-1 expression of the heart in response to diabetes and myocardial IR injury.

Although it is well known that diabetes contributes to coronary artery spasm and myocardial ischemia in patients, the pathophysiologic role of BAG3 on resistant arterioles and cardiomyocytes has not yet been determined. We hypothesized that diabetes may impair the function and molecular mechanism of coronary arteries and cardiomyocytes to exaggerate further oxidative injury in the heart. Mesenteric arterioles and coronary arteries are both resistant arterioles and have a similar diameter and a close pathophysiological regulation during ischemia. Therefore, we first examined the diabetes effect on BAG3 expression and the vasoreactivity of mesenteric arterioles in response to vasoconstrictors or vasodilators. In the heart, we adapted a novel chemiluminescence (CL) method for the direct measurement of the amount of ROS produced from the heart surface and determined the possible cellular location of de novo ROS synthesis in the diabetic and/or IR heart. Finally, our study explored the response of the cardiac endogenous defense mechanism in BAG3/Bcl-2/Nrf-2/HO-1 and NADPH oxidase p22/p67-mediated oxidative stress in response to diabetes and myocardial IR injury.

## 2. Methods and Materials

### 2.1. Animals

Male Wistar rats (200–250 g) were obtained from BioLASCO Co. Ltd. (I-Lan, Taiwan) and housed at the Experimental Animal Center, National Taiwan Normal University, at a constant temperature and with a consistent light cycle (light from 07:00 to 18:00 o’clock). The rats were fed standard chow and tap water ad libitum. All surgical and experimental procedures were approved by the Institutional Animal Care and Use Committee of National Taiwan Normal University with the Approval number 10732 and followed the guidelines of the National Science Council of Republic of China.

### 2.2. Grouping

The detailed grouping protocol was indicated below. Four groups of rats (*n* = 10 in each group) were used: (1) control (CON) group treated with sham operation; (2) streptozotocin (STZ)-treated group with sham operation and intraperitoneal STZ treatment; (3) control rats with myocardial ischemia/reperfusion (IR CON) injury group and (4) diabetic rats with myocardial ischemia/reperfusion injury (IR STZ). On the experimental day for cardiac hemodynamic evaluation, an anesthetized rat with a baseline heart rate > 300 beats/min and left ventricular systolic pressure > 100 mmHg was adapted for the study. In this study, 2 STZ rats died during diabetes induction, whereas 2 IR CON rats and 3 IR STZ rats died during myocardial IR induction. We have excluded these rats in our study.

### 2.3. Type I Diabetes Induction

The protocol for induction of type I diabetes was demonstrated previously [25]. We used a single dose of intraperitoneal streptozotocin administration (STZ at 65 mg/kg; Sigma, Missouri, KC, USA) to destroy pancreatic β cells. Eight-week-old rats were fasted 8 h before and under 2% isoflurane, the rats were treated with STZ in 10 mg/mL of 0.1 M sodium citrate buffer (pH 4.5; Sigma, Missouri, KC, USA). We determined and recorded the body weight and fasted blood glucose before and after STZ treatment every week. After 8 weeks of STZ treatment, the fasted (8 h) blood glucose level was examined by using the Contour plus blood glucose monitoring system (Bayer, Leverkusen, Germany). Fasted blood glucose > 250 mg/dL was identified as successful diabetes induction.

### 2.4. Metabolic Cage Analysis

We determined 24 h of food and water intake and urine and feces weight by a metabolic cage.

### 2.5. Evaluation of Cardiac Hemodynamic Parameters

Under urethane (1.2 g/kg, Sigma, Missouri, KC, USA, intraperitoneally), the trachea of the rat was intubated for artificial ventilation (Small Animal Ventilator Model 683) with 50 breaths/min, a tidal volume of 8 mL/kg and a positive end-expiratory pressure of 5 cm H_2_O. Hemodynamic examination was performed as previously described [26]. Hemodynamic parameters in the left ventricle, including the heart rate (HR), left ventricular end-diastolic pressure (LVEDP), left ventricular systolic pressure (LVSP), left ventricular pressure (LVDP = LVSP − LVEDP), peak rate of increase or decrease in left ventricular pressure (±dp/dt), were determined by a PE50 tubing containing heparinized saline with a pressure transducer introduced into the left ventricle through the right carotid artery. As indices of contractility and relaxation in the left ventricular function, the maximal rates of +dP/dt and −dP/dt were determined in 10 CON IR and 10 STZ IR rats.

All the parameters were simultaneously recorded with an iWorx 214 data recorder (IX-214; iWorx Systems, Inc., WA, USA). After the assessment of the basal parameters, myocardial infarction was performed to assess the hemodynamic parameters at the stages of ischemia and reperfusion. At least 6 consecutive cardiac cycles were measured for each animal. A Powerlab data acquisition system (ADI Instuments) was used for acquiring data throughout the experiment.

### 2.6. Induction of Myocardial Ischemia/Reperfusion

Under anesthesia and ventilation with Small Animal Ventilator Model 683 (Harvard Apparatus, Holliston, MA, USA), midline sternotomy was performed. The left anterior descending artery, approximately 3 mm away from the left coronary ostium, was ligated with 6-O Prolene (Ethicon Inc., Somerville, NJ, USA) and a slipknot was tied to develop reversible coronary arterial occlusion as described before [27]. After 45 min of ischemia, the slipknot was released, and the heart was reperfused for 4 h in 10 CON IR and 10 STZ IR rats.

### 2.7. Heart Microcirculation Determination

To determine the in vivo and real-time response of coronary arterial constriction on cardiac surface microcirculation, we used a full-field laser perfusion imager (MoorFLPI, Moor Instruments Ltd., Devon, UK) to continuously record the cardiac microcirculatory blood flow intensity as described previously [28]. Briefly, the imager utilized laser speckle contrast imaging to display the random speckle pattern. The random speckle pattern alters when blood cells enter into the region of interest (ROI). The contrast image is processed to obtain a 16-color-coded image. For example, the blue color set at 0 perfusion unit (PU) is indicated as a low blood flow and the red color set at 1000 PU as a high blood flow. The microcirculatory blood flow intensity of each ROI was displayed as Flux with perfusion unit in the selected area. The PUs were assayed by the MoorFLPI software version 3.0 (MoorFLPI, Moor Instruments Ltd., Devon, UK).

### 2.8. In vivo Real-Time Chemiluminescence Recording of Heart Superoxide Anion Activity

We determined the whole heart ROS in response to myocardial ischemia/reperfusion in vivo via an intravenous administration of a superoxide anion probe, 2-Methyl-6-(4-methoxyphenyl)-3,7-dihydroimidazo-[1,2-a]-pyrazin-3-one-hydrochloride (MCLA) (0.2 mg/mL/h, TCI-Ace, Tokyo Kasei Kogyo Co. Ltd., Tokyo, Japan, and recorded with an ultrasensitive Chemiluminescence Analyzing System (CLA-ID3, Tohoku Electronic In. Co., Sendai, Japan), as described previously [27]. In brief, the anesthetized rat maintained by a respirator and a circulating water pad at 37 °C during determination. For excluding photon emission from sources other than the heart, the anesthetized animal was placed in a dark box with a shielded plate. Only the heart was unshielded and positioned under a reflector to reflect the MCLA-enhanced chemiluminescent signals from the heart surface onto the detector area. The total chemiluminescent ROS level was measured by area under curve from the heart. The chemiluminescent signal obtained from 0.2 mL saline in 1 mL of MCLA (0.2 mg/mL) or 0.2 mL xanthine (0.75 mg/kg body weight)/xanthine oxidase (24.8 mU/kg body weight) in 1 mL of MCLA (0.2 mg/mL) was designed as a negative or positive control as described previously [27].

### 2.9. Mesenteric Arterial Wire Myography

We applied the wire myographic method to investigate the mesenteric reactivity to various vasoactive agents as previously described [28]. We used the isolated second-order mesenteric artery (~ 150 μm) [29], which is similar to the coronary artery (~ 160 μm) with a similar-diameter arteriole [13]. These arterioles were equilibrated for 30 min, and tested for vasoreactivity using norepinephrine (NE) and acetylcholine (Ach). Vasoconstriction in response to NE (10^−9^~10^−3^ mol/L) and vasorelaxation in response to Ach (10^−9^~10^−3^ mol/L) were measured.

### 2.10. Western Blot

The expression of β-actin (#4970, 1:1000; Cell Signaling Technology, MA, USA), 4-HNE (bs-6313R, 1:1000; Bioss, MA, USA), BAG3 (NBP2-27398, 1:2000; Novus, CO, USA), Bcl-2 (bs-0032R, 1:1000; Bioss, MA, USA), caspase 3 (bs-0081R, 1:1000; Bioss, MA, USA), p-eNOS (ser1177, 1:1000; Cell Signaling Technology, MA, USA) and PARP (#9532, 1:1000; Cell Signaling Technology, MA, USA) from the damaged heart infarct areas after ischemia/reperfusion injury were evaluated by Western blotting as described before [26]. The data were recorded and analyzed with a luminescent image analyzer (ImageQuant LAS 4000 mini; GE Healthcare, Little Chalfont, UK).

### 2.11. Infarct Size Calculation

Infarct size was measured as described previously [26]. In brief, after IR, 5 mL methyl blue was injected via the jugular vein and then the heart was removed. The heart was serially sliced from base to apex at 3-mm intervals. These slices were incubated at 37 °C for 20 min in 2% 2,3,5-triphenyltetrazolium chloride (TTC; Sigma–Aldrich) to stain the infarct (white) and the viable (red) area. The cardiac slice images were taken with a digital camera (Nikon, Tokyo). The infarct area was estimated with Image-Pro Plus 6.0 (IPP 6.0; Roper Industries, Sarasota, FL, USA).

### 2.12. In situ Demonstration of Fibrosis, Oxidative Stress and Apoptosis Formation

We compared the degree of oxidative stress by 4-HNE and apoptosis by poly(ADP-ribose) polymerase (PARP) and terminal deoxynucleotidyl transferase-mediated dUTP-biotin nick end labeling (TUNEL) staining. Histochemical staining was performed on formalin-fixed, paraffin-embedded heart sections with or without IR injury by haematoxylin and eosin, and Masson’s trichrome stain for the detection of collagen deposits (fibrosis), 4-HNE (bs-6313R, 1:200; Bioss, MA, USA), BAG3 (NBP2-27398, 1:1000; Novus, Colorado, USA), Bcl-2 (bs-0032R, 1:100; Bioss, MA, USA), cleaved-caspase 3 (#9661, 1:300; Cell Signaling Technology, Mass, USA), rabbit monoclonal PARP (ab191217, 1:500, Abcam’s RabMb^®^ technology, Cambridge, UK) and TUNEL stains (BioVision, CA, USA). Each section was incubated with the respective primary antibody overnight at 4 °C. After washing, the section was incubated with Envision system horseradish peroxidase-labeled polymer (Dako, Glostrup, Danemark for 1 h at room temperature. Apoptotic cells in the heart were determined with TUNEL stains [27]. The number of TUNEL positive cells was counted at 20 high-power fields (×400). The percentage of positive stain area in the brown color of PARP was analyzed in the section area. Twenty high-power (×400) fields were randomly selected for each section, and the level of each oxidative stress was analyzed with a Sonix Image Setup (Sonix Technology Co., Ltd., Hinschu, Taiwan).

### 2.13. MDA Assay

We determined MDA concentration by using Lipid Peroxidation (MDA) Assay Kit (ab118970; BioVision, CA, USA). The method of MDA determination was according to the manufacture process.

### 2.14. Statistical Analysis

We used GraphPad Prism 6 (GraphPad Software, CA, USA) to perform data analysis. All values were expressed as mean ± standard error mean. Data were subjected to one-way analysis of variance (ANOVA), followed by Duncan’s multiple-range test for assessment of the difference among groups. Differences within groups were evaluated by paired *t* test. Differences were regarded as significant if *p* < 0.05 was attained.

## 3. Results

### 3.1. STZ Induced Type I Diabetes Led to Decreased Body Weight and Hyperglycemia

**Figure 1:**
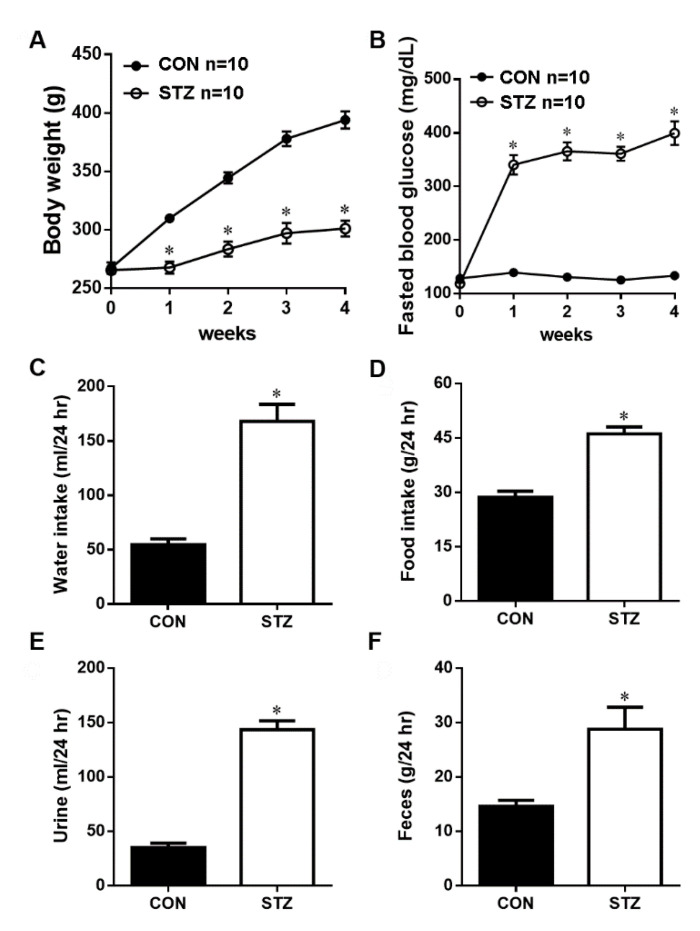
The basal physiologic parameter of body weight (**A**), fasted blood glucose (**B**), water intake (**C**), food intake (**D**), urine amount (**E**) and feces amount (**F**) in the control (CON) and streptozotocin-diabetic (STZ) rats. After 4 weeks of STZ induction, these physiological parameters are significantly increased as compared with CON rats. Data are expressed as mean ± SEM (*n* = 10) using the single values. * *p* < 0.05 vs. CON.

We confirmed that intraperitoneal STZ destroyed pancreatic β cells, significantly (*p* < 0.05) decreased body weight (Figure 1A) and elevated fasted blood glucose (*p* < 0.05, Figure 1B) after one week of STZ induction vs. CON rats.

### 3.2. STZ Induced Type I Diabetes Evoked Polydipsia, Polyuria and Polyphagia

We evaluated the effect of four weeks of STZ-induced type I diabetes on several physiological parameters in the 24 h metabolic cage analysis. Our data showed that during 24 h of metabolic cage data, the water intake (Figure 1C), food intake (Figure 1D), urine volume (Figure 1E) and feces weight (Figure 1F) were all significantly (*p* < 0.05) increased as compared with the CON group.

### 3.3. Diabetes Affects Mesenteric Arterial Proteins Expression and Responses to Vasoactive Agents

**Figure 2:**
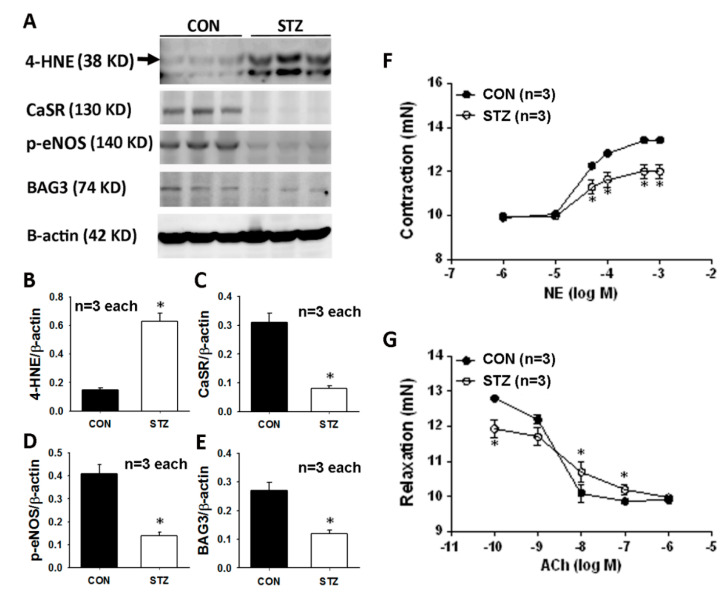
Mesenteric vascular Western blot graph (**A**) and analysis of 4-HNE (**B**), CaSR (**C**), p-eNOS (**D**) and BAG3 (**E**) are demonstrated. Microvascular reactivity from mesenteric arterioles to a vasoconstrictor (norepinephrine, NE, **F**) and vasodilator (acetylcholine, Ach, **G**) is displayed in CON and STZ groups. All data are presented as the mean ± SEM (*n* = 3) using the single values in each test. * *p* < 0.05 vs. CON group.

We explored the mesenteric arterial 4-HNE, CaSR, p-eNOS and BAG3 expression in the CON and STZ rats. The original graph of the Western blot is displayed in Figure 2A. The significantly upregulated 4-HNE expression (Figure 2B) and downregulated CaSR (Figure 2C), p-eNOS (Figure 2D) and BAG3 (Figure 2E) were found in the STZ mesenteric arterioles. 

We determined microvascular responses to NE and Ach in a secondary branch of mesenteric arteries (MAs) in the diabetic rats after eight weeks of STZ induction. As shown in Figure 2F, the presence of NE ≧ the dose of 3 × 10^−5^ M resulted in a significant decrease (*p* < 0.05) in vasoconstriction in STZ rats compared with CON rats. When the MAs were treated with the Ach, all CON MAs displayed vasodilation to different Ach stimulation (Figure 2G). Under the concentration of 10^−10^ M of Ach, the vasodilatory level of STZ MAs was significantly higher than that in CON MAs. However, under higher concentrations of 10^−8^~10^−7^ M of Ach, relaxant responses in MAs were significantly attenuated in STZ rats compared with CON rats.

### 3.4. Diabetes Exacerbated Myocardial IR Induced Left Ventricular Dysfunction

**Figure 3:**
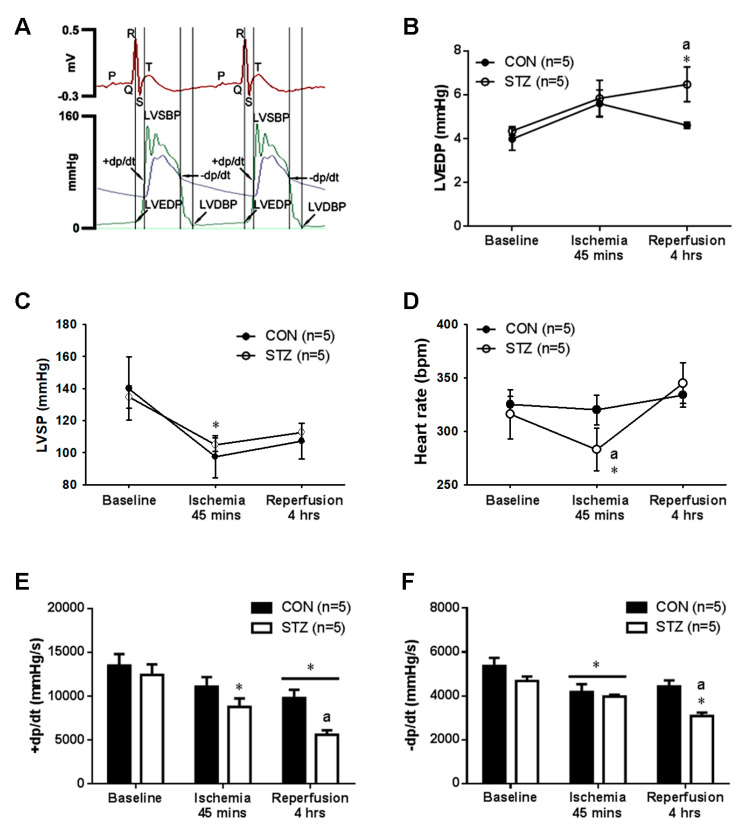
The myocardial ischemia/reperfusion (IR) effect on several cardiac parameters in CON and STZ rats. The original graphs on left ventricular pressure tracing are shown in one CON rat (**A**). In response to myocardial IR injury, the increased left ventricular end-diastolic pressure (LVEDP) (**B**) during the reperfusion stage, the decreased left ventricular systolic pressure (LVSP) (**C**) and heart rate (**D**) during the ischemic period and the decreased value of +dp/dt (**E**) and −dp/dt (**F**) are noted in the 5 STZ rats as compared with the 5 CON rats. * *p* < 0.05 vs. CON, a *p* < 0.05 vs. STZ.

As reported previously [26], we successfully developed a technique for the direct measurement of cardiac hemodynamics including LVSBP, LVEDP and ±dp/dt in the anesthetized rats (Figure 3A). The baseline level of LVEDP (Figure 3B), LVSBP (Figure 3C), heart rate (Figure 3D) and the calculated +dp/dt (Figure 3E) and −dp/dt (Figure 3F) were all similar between CON and STZ groups. In response to IR injury, the significant elevation in LVEDP (Figure 3B) during the reperfusion period, the significant decrease in LVSP (Figure 3C) and heart rate (Figure 3D) during the ischemic stage and the decrease in +dp/dt (Figure 3E) and −dp/dt (Figure 3F) were found in IR STZ rats as compared with IR CON rats. The decrease in heart rate and LVSP and the increase in LVEDP impaired the left ventricular contractile function.

### 3.5. Diabetes Exacerbated Oxidative Stress and Depressed Antioxidant Defense in the Heart

**Figure 4:**
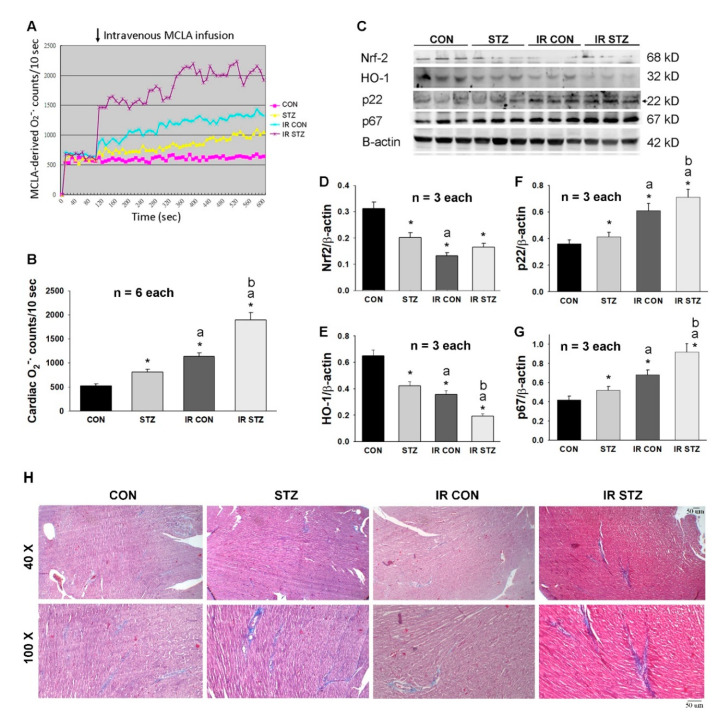
The diabetes effect on IR affected cardiac O_2_^−^ amount (**A**,**B**, *n* = 6), antioxidant defense mechanism, Nrf-2 and HO-1 expression (**C**,**D**,**E**, *n* = 3) and NADPH oxidase p22 and p67 expression (**C**,**F**,**G**, *n* = 3) using the single values in four groups of rats. Data are expressed as mean ± SEM. The Masson’s trichrome stain (yellow head) for cardiac fibrosis is indicated in **H**. * *p* < 0.05 vs. CON, a *p* < 0.05 vs. STZ, b *p* < 0.05 vs. IR CON.

We determined the effect of STZ-induced diabetes and myocardial IR on oxidative stress and the antioxidant defense mechanism in hearts by Western blotting. Our data from the in vivo cardiac CL determination technique (Figure 4A) demonstrated that cardiac ROS were displayed in an order: IR STZ > IR CON > STZ > CON (Figure 4B). We speculated that the antioxidant defense mechanism of Nrf-2/HO-1 signaling may be depressed during diabetes and IR injury. Our data found that Nrf-2 and HO-1 expression was significantly decreased in STZ, IR CON and IR STZ hearts as compared with CON hearts (Figure 4C–E). We further explored the possible sources of oxidative stress of NADPH oxidase p22 and p67 expression in these four groups of hearts. NADPH oxidase p22 and p67 expression was significantly increased in STZ, IR CON and IR STZ hearts as compared with CON hearts (Figure 4C,F,G). We further determined the fibrosis degree by Masson’s trichrome stain in these four groups of hearts. As shown in Figure 4H, the markedly increased blue color of Masson’s trichrome stain was noted in the STZ and IR STZ hearts, indicating diabetes enhanced cardiac fibrosis.

### 3.6. Diabetes Further Exacerbated Myocardial IR Evoked Oxidative Stress, Inflammation and Injury

**Figure 5:**
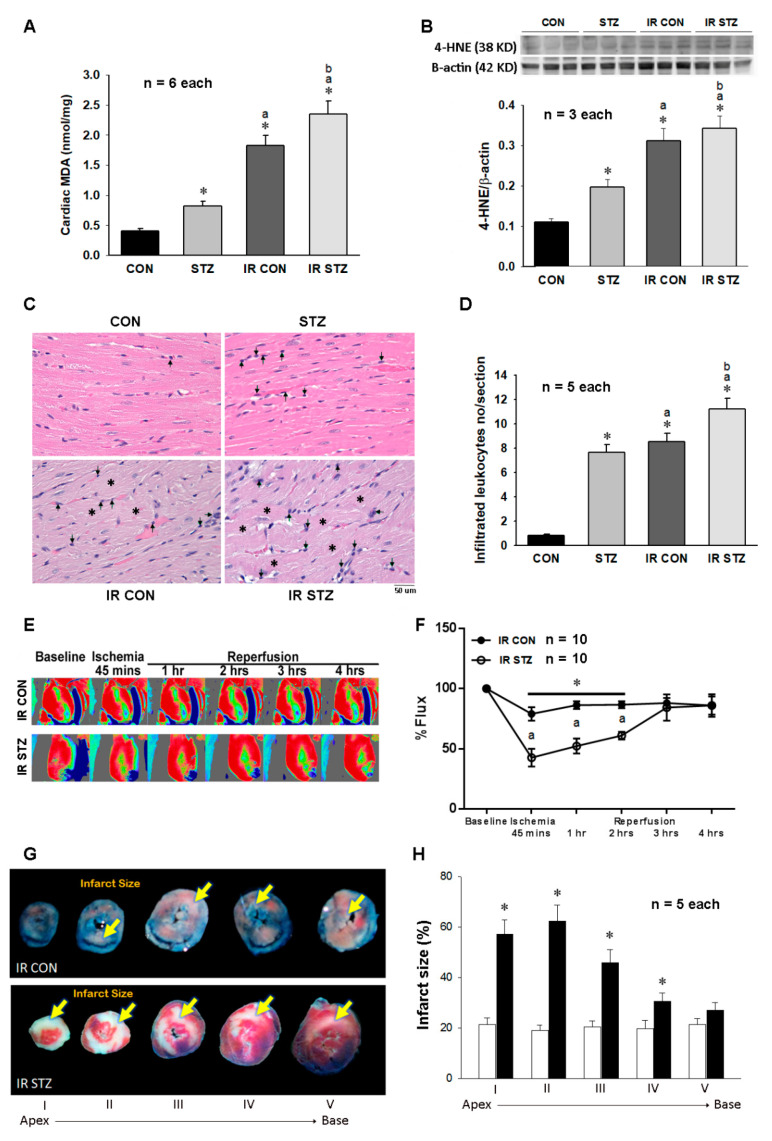
The diabetes effect on IR affected cardiac lipid peroxidation in MDA content (**A**, *n* = 6), 4-HNE expression (**B**, *n* = 3), leukocyte infiltration and cardiac structure (**C**,**D**, *n* = 5), microcirculation (**E**,**F**, *n* = 10) and infarct size (**G**,**H**, *n* = 5) in IR CON and IR STZ rats. Data are expressed as mean ± SEM. In graph C, the arrow indicates the infiltrated leukocyte and the star indicates cardiac structural alteration. * *p* < 0.05 vs. CON, a *p* < 0.05 vs. STZ.

As shown inFigure 5A, we investigated the amount of the lipid peroxidation product MDA in the four groups of rat hearts. Our data indicated that the cardiac MDA concentration was consistent with the ROS data in Figure 4B and also displayed in an order: IR STZ > IR CON > STZ > CON (Figure 5A), implicating diabetes exacerbates oxidative stress. A consistent finding for MDA was also obtained in the cardiac 4-HNE expression, which was expressed in the order: IR STZ > IR CON > STZ > CON (Figure 5B). According to H&E stain, the increased leukocyte infiltration number was found in STZ, IR CON and IR STZ as compared with CON hearts (Figure 5C). The statistical data demonstrated the leukocyte infiltration number in an order of IR STZ > IR CON > STZ > CON (Figure 5D).

The baseline level of hearts in CON and STZ hearts displayed a similarly red color intensity (Figure 5E). In response to ischemia, CON and STZ hearts both markedly displayed a decreased blood perfusion to a green to blue color in the lower region of the left ventricle. However, during the ischemic phase and reperfusion periods at 1 and 2 h, the microcirculation in the selected area of the IR STZ hearts was significantly lower as compared with IR CON hearts (Figure 5F). The myocardial infarct size of the five sections was observed in IR CON and IR STZ hearts (Figure 5G). It showed that infarct size was markedly increased in both IR CON and IR STZ hearts. However, the infarct area was significantly increased in IR STZ hearts as compared with IR CON hearts (Figure 5H).

### 3.7. Diabetes Exacerbated Myocardial IR Depressed Cardiac BAG3 Expression

**Figure 6:**
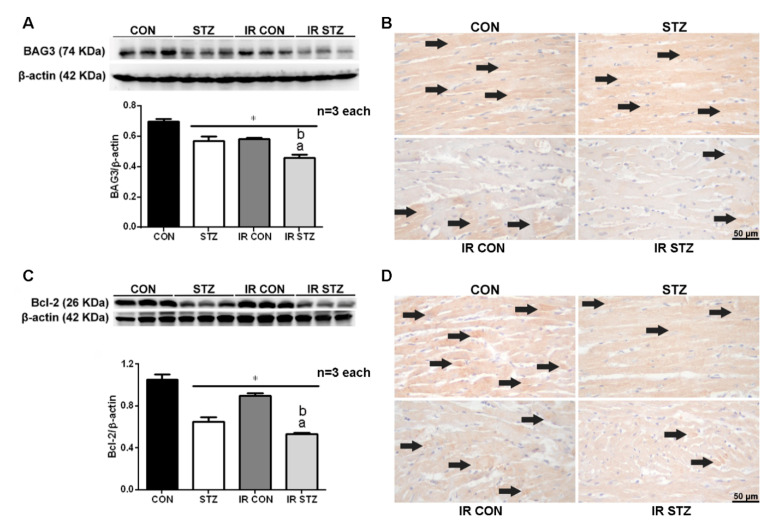
Western blot and immunohistochemistry of BAG3 (**A**,**B**) and Bcl-2 (**C**,**D**) in IR myocardium. **BAG3** and Bcl-2 expression is significantly decreased in the STZ group than those in the CON group. The BAG3 and Bcl-2 expression is also significantly reduced in the IR CON and IR STZ groups as compared with the CON group. IR STZ hearts further depress BAG3 and Bcl-2 expression than IR CON hearts. Data are expressed as mean ± SEM (*n* = 3) using the single values. * *p* < 0.05 vs. CON, a *p* < 0.05 vs. STZ, b *p* < 0.05 vs. IR CON.

We explored the effect of STZ-induced diabetes and myocardial IR on cardiac BAG3 expression by Western blotting and immunohistochemistry. Our data showed that cardiac BAG3 expression was significantly decreased in STZ compared with CON hearts (Figure 6A, *p* < 0.05). Myocardial IR significantly depressed cardiac Bcl-2 expression in both IR CON and IR STZ groups compared with CON hearts. Furthermore, the cardiac BAG3 expression was further decreased in IR STZ than that in the STZ or IR CON group (Figure 6A, *p* < 0.05). The BAG3 immunohistochemistry (Figure 6B) also demonstrated a consistent expression with the Western blot data.

### 3.8. Diabetes Exacerbated Myocardial IR Depressed Cardiac Bcl-2 Expression

We determined the effect of STZ-induced diabetes and myocardial IR on cardiac Bcl-2 expression by Western blotting and immunohistochemistry. Our data showed that the Bcl-2 expression was significantly decreased in STZ compared with CON hearts (Figure 6C, *p* < 0.05). Myocardial IR significantly depressed cardiac Bcl-2 expression in both IR CON and IR STZ groups vs. CON hearts. The cardiac Bcl-2 expression was further decreased in IR STZ than that in the STZ or IR CON group (Figure 6C, *p* < 0.05). The Bcl-2 immunohistochemistry (Figure 6D) also displayed a consistent finding with the Western blot data.

### 3.9. Diabetes Exacerbated Myocardial IR Enhanced Apoptosis Formation

**Figure 7:**
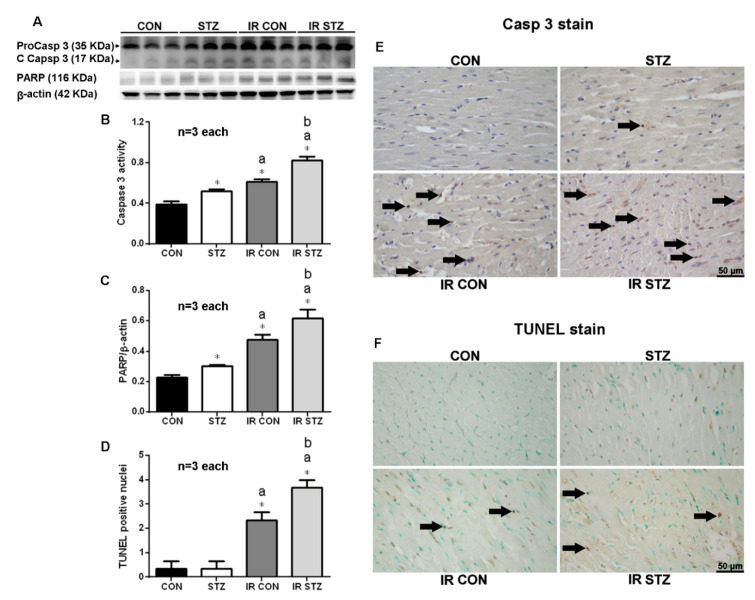
Western plot (**A**) and immunohistochemistry (**E–F**) of myocardial caspase 3, and PARP and apoptosis TUNEL stain in IR myocardium. The statistic data in Caspase 3 (**B**), PARP (**C**) and TUNEL positive nuclei (**D**) are indicated. Data are expressed as mean ± SEM (*n* = 3) using the single values. * *p* < 0.05 vs. CON. a *p* < 0.05 vs. STZ. b *p* < 0.05 vs. IR CON.

We determined the effect of STZ-induced diabetes and myocardial IR-evoked apoptosis by Western blotting and immunohistochemistry from the infarct area. Our data showed that the cardiac cleaved-caspase 3 expression was significantly increased in the STZ compared with the CON group (Figure 7A, *p* < 0.05). Myocardial IR significantly enhanced cardiac cleaved-caspase 3 expression in both IR CON and IR STZ groups vs. CON hearts. The cardiac cleaved-caspase 3 expression was further upregulated in IR STZ than that in the STZ or IR CON group (Figure 7A, *p* < 0.05). The cardiac cleaved-caspase 3 immunohistochemistry (Figure 7B) also displayed a consistent finding with the Western blot data.

We further examined the effect of STZ-induced diabetes and myocardial IR-evoked PARP expression by Western blotting (Figure 7A). Our data showed that the cardiac PARP expression was significantly increased in the STZ compared with the CON group (Figure 7C, *p* < 0.05). Myocardial IR significantly enhanced cardiac PARP expression in both IR CON and IR STZ groups vs. CON hearts. The cardiac PARP expression was further upregulated in IR STZ than that in the STZ or IR CON group (Figure 7C, *p* < 0.05). The cardiac TUNEL stain performed by immunohistochemistry is indicated in Figure 7F. The statistic data from TUNEL immunohistochemistry also displayed a consistent result with the Western blot data of caspase 3 (Figure 7B) and PARP (Figure 7C).

## 4. Discussion

Our physiologic data showed that the STZ-induced diabetes model was successfully characterized by decreased body weight, hyperglycemia, polydipsia, polyuria and polyphagia. Our data evidenced that STZ-induced type 1 diabetes increased oxidative stress, depressed BAG3, Bcl-2, CaSR and p-eNOS expression and evoked vascular dysfunction in vasoconstriction and vasodilation of the mesenteric arterioles. Diabetes also increased cardiac ROS, NADPH oxidase p22/p67-mediated oxidative stress, MDA and 4-HNE expression and leukocyte infiltration in the cardiac tissue. Diabetes further exacerbated myocardial IR-induced left ventricular dysfunction and infarct size, and the depression of cardiac microcirculation through the enhancement of leukocyte infiltration and NADPH oxidase p22 and p67 derived ROS and oxidative stress and a reduction in BAG3, Bcl-2 and Nrf-2/HO-1 protective signaling in the heart.

Vasoreactivity examination has been widely performed in isolated rat aortic rings [30]. However, such vascular responses in the aorta cannot be generalized to other vascular beds, thus we explored the influences of diabetes on resistance arteries like mesenteric arterioles because these vessels actually regulate arterial blood pressure and organ blood flow by the mechanism of vasoconstriction and vasodilation. Mesenteric arterioles were applied to examine the vascular reactivity to vascular agents for several reasons as described before [28]. First, mesenteric arterioles are more easily obtained than coronary arteries. Second, coronary and mesenteric arterioles have a similar diameter and are resistant arteries. Third, mesenteric ischemia is often associated with coronary artery stenosis, suggesting their close pathophysiological regulation. In addition, the advantages of the application of mesenteric arteries over radial arteries or venous conduits are due to a lower vasospasm and higher patency rate [31]. In spite of several differences in microvascular function and sensitivity between mesenteric and coronary arterioles, these include a differential blood supply, metabolic demand and response to blood flow regulation [32]. The use of mesenteric arterioles can provide some important information like coronary artery.

Endothelial dysfunction includes a reduced endothelium-dependent vasorelaxation to the vasodilators, like Ach stimulation or flow-mediated vasodilation. Zhang et al. [33] observed that diabetes impaired mesenteric endothelium-dependent vasorelaxation through the loss of endothelium-derived hyperpolarizing factor, decreased sensitivity of smooth muscle to NO and/or decreased NO bioavailability rather than reduced basal NO production. In both male and female rats, diabetes also decreased the vasoconstrictive activity in mesenteric arteries possibly through the alteration to α1-adrenoceptor [34]. Our mesenteric wire myograph data with an alteration to the vasoconstriction and vasodilation of diabetic mesenteric arterioles were consistent with the findings from Zhang et al. [33] and Park et al. [34]. Our data further showed that the enhanced 4-HNE oxidative stress and the downregulated BAG3, CaSR and p-eNOS were identified in the diabetic mesenteric vascular beds. Some biomarkers in NO signaling are indicated in response to myocardial IR [35]. For example, our previous results demonstrated that decreased eNOS expression and NO levels were observed in the markedly reduced cardiac microcirculation [28]. CaSR is expressed in endothelial and smooth muscle cells, and through extracellular Ca^2+^ stimulation, CaSR is activated to trigger a NO-dependent relaxation [23]. During metabolic stress like diabetes, the impaired CaSR activity may contribute to the macrovascular late complications [23]. Our data in mesenteric arterioles observed that CaSR expression was depressed in diabetes suggesting an impairment of Ca^2+^-mediated relaxation in diabetic vessels.

We hypothesized that the increased oxidative stress by excess ROS production and/or depressed endogenous antioxidant defense mechanism mainly contributed to cardiovascular dysfunction and injury. In diabetes, hyperglycemia enhanced cardiac oxidative stress to further exacerbate cardiac IR injury and dysfunction [36]. STZ-induced diabetes increased cardiac oxidative stress such as the elevated isoprostane amount and the depressed plasma SOD activity associated with the increased TNFα expression and the decreased NO amount, whereas myocardial IR further enhanced these oxidative stresses [13]. Our data evidenced further increased ROS, NADPH oxidase p22/p67, 4-HNE and MDA in the diabetic hearts subjected to IR injury. Oxidative stress decreased the availability of NO and contributed to the loss of protective function in response to cardiac IR injury [37]. Under diabetes and ischemic conditions, large amounts (μM) of NO production, a toxic and damaging agent, by the exacerbated activation of inducible nitric oxide synthase led to severe cardiomyocyte injury [38].

There are two important upstream signaling pathways, PI3K/Akt and JAK2/STAT3, playing a critical role in the enforcement of myocardial NO availability. AKT activation would confer cellular protection via the downstream of eNOS and Bcl-2. STAT3 also confers an independent AKT cardiac protective mechanism via the upregulation of anti-Bcl-xl and Bcl-2 [36]. In diabetic hearts, cardiac IR injury exacerbated cardiac PTEN expression and this overexpression of PTEN impaired the PI3K/Akt and JAK2/STAT3 protective signaling pathways in the IR heart [39]. Our data and previous finding [40] consistently demonstrated that diabetic hearts depressed BAG3 and Bcl-2 expression, which promoted myocardial apoptosis, increased infarct size, induced irregular arrangement of cardiomyocytes and pathological changes and elevated LVEDP after myocardial IR injury. Our data also found a decrease in Nrf-2/HO-1 signaling in the diabetic hearts and a further depression of Nrf-2/HO-1 signaling in response to IR injury. A depressed endogenous defense mechanism may contribute to diabetic cardiomyopathy and myocardial IR injury.

Cardiac IR injury increased excess ROS formation from the cardiomyocytes and endothelial cells and the increased ROS impaired DNA and activated PARP, leading to damage and cell death of cardiomyocytes and endothelial cells [41]. Diabetes through NADPH oxidase p22 and p67 mediated oxidative stress and inflammatory response contributed to diabetic cardiomyopathy and cardiac dysfunction [42]. The data using targeted gene disruption in mice or BAG3-deficient mice to examine BAG3 function indicated deficiency in BAG3 would develop severe myopathy in Z-disk architecture and myofibrillar apoptosis implicating BAG3 contributes to maintaining the myofibrillar structure [20]. BAG3 and Bcl-2 interacted with each other and played the anti-apoptotic action [17]. BAG3 via autophagy and lysosome interacted with mitochondria to remove the damaged mitochondria and the decreased BAG3 expression would significantly reduce autophagy formation, lead to the accumulation of damaged mitochondria and enhance apoptosis production [21]. Myocardial IR decreased mice cardiac BAG3 and Bcl-2 and upregulated cleaved-caspase 3 expression, whereas treatment of mice with BAG3 before IR efficiently reduced infarct size and recovered left ventricular function and inhibited autophagy and apoptosis [12]. STZ decreased Nrf-2/HO-1 expression and induced inflammation, oxidative stress, fibrosis and apoptosis in the diabetic cardiomyopathy, leading to cardiac dysfunction and impairment [16]. Our present data evidenced that diabetes decreased BAG3, Bcl-2, Nrf-2 and HO-1 expression, further reduced myocardial IR-depressed BAG3, Bcl-2, Nrf-2 and HO-1 expression and increased cleavage caspase 3/PARP, leading to apoptosis formation in the heart. Moreover, diabetes exacerbated myocardial IR injury and left ventricular dysfunction characterized by further depressed microcirculation, heart rate, left ventricular systolic pressure and peak rate of pressure increase/decrease (±dp/dt) and increased LVEDP and infarct size in diabetic hearts. Our data informed us that the impairment in cardiac function by diabetes-induced oxidative stress further weakens the diastolic and systolic capabilities of the heart.

## 5. Conclusions

**Figure 8:**
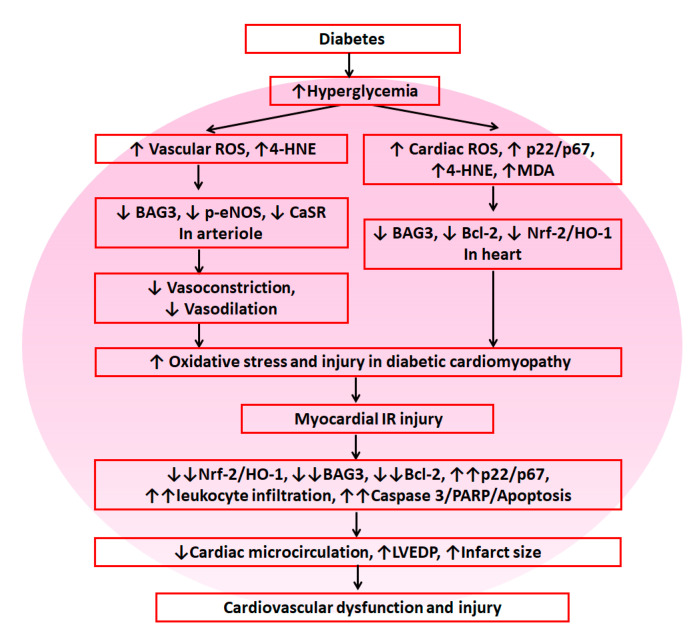
The summary diagram is demonstrated. Diabetes enhanced cardiovascular damage by suppression of antioxidant defense mechanisms including Nrf-2/HO-1, BAG3 and Bcl-2 and increased oxidative stress including vascular and cardiac reactive oxygen species (ROS). In response to myocardial IR injury, diabetes further decreased antioxidant defense mechanisms and enhanced oxidative stress/injury, leading to cardiovascular dysfunction and injury.

As shown in Figure 8, these results suggest that diabetes exacerbates myocardial IR-induced dysfunction and injury through the upregulation of NADPH oxidase p22- and p67-mediated oxidative stress and downregulation of Nrf-2/HO-1, BAG3 and Bcl-2 in the myocardium, leading to further cardiac dysfunction and cardiovascular injury. According to our data, reductive stress did not play a role in diabetes-induced myocardial IR injury.

## Abbreviations

4-HNE	4-hydroxynonenal
ACh	acetylcholine
BAG3	Bcl-2-associated athanogene 3
Bcl-2	B-cell lymphoma 2
+dp/dt	peak rate of pressure increase
−dp/dt	peak rate of pressure decrease
Drp1	dynamin-related protein 1
eNOS	endothelial nitric oxide synthase
GSK-3β	glycogen synthase kinase-3 beta
GULT	glucose transporter
H&E stain	hematoxylin and eosin stain
IR	ischemia reperfusion
JAK2	Janus kinase 2
LVEDP	left ventricular end-diastolic pressure
MDA	malondialdehyde
NE	norepinephrine
PARP	poly (ADP-ribose) polymerase
PI3K	phosphoinositide 3-kinase
PTEN	phosphatase and tensin homologue deleted on chromosome 10
ROS	reactive oxygen species
Sirt1	sirtuin1
STAT3	signal transducer and activator of transcription 3
STZ	streptozotocin
TUNEL	terminal deoxynucleotidyl transferase dUTP nick end labeling
